# Pathology in Surgical Practice

**Published:** 1986-10

**Authors:** J. D. Davies


**PATHOLOGY IN SURGICAL PRACTICE**

**Eds G. J. Hadfield, M. Hobsley and B. C. Morson**
**Edward Arnold, London**
**pp. 500, hardback, with a Foreward by the Presidents of the Royal Colleges of Surgeons and of Pathologists**
**ISBN 0 7131 4471 8. Price £49.50**

Some books are like children; a few have an eminently respectable parentage. This book is obviously high-born. The presidents of the Royal Colleges of Surgeons and Pathologists clearly both think that it is a good thing for surgeons to talk to their pathological colleagues, and *vice versa*.

The aim of this book is to improve communication between surgeons and histopathologists. It reflects the 'team' approach to clinical management, and takes the form of a series of chapters on 35 surgical topics. Each chapter is written by a pair of surgeons and pathologists, most of whom work in the same hospital.

Probably the most illuminating section is the editorial introduction, in which the basis of modern clinico-pathological communication is outlined. This chapter contains many useful pieces of advice to both surgeon and pathologist. The need for adequate clinical information on the request form, and the virtues of brevity in the pathological report are clearly indicated. In addition the usefulness of periodic review meetings is emphasised. Subjects less often considered include the instruction of theatre nursing staff in the handling of specimens, and the possibilities of more frequent specimen photography. After decades of neglect, the specimen 'pot' also makes a come-back here. There is much to absorb in this excellent introductory chapter.

The chapters on the surgical topics are generally informative, but there are minor blemishes. The statement on p. 264 that 'Kaposi sarcoma(s) are malignant tumours which arise in oedematous limbs' is best forgotten. Unfortunately the decision was taken not to incorporate references in the body of the text, and merely to attach lists of further reading to the individual chapters. The index has its deficits; for example there is no mention of Diabetes insipidus or mellitus.

However this book does seem to be a useful addition to the publications on Surgical Pathology. It should do much to help pathologists and surgeons understand each others' problems.

J. D. Davies

